# FQSqueezer: *k*-mer-based compression of sequencing data

**DOI:** 10.1038/s41598-020-57452-6

**Published:** 2020-01-17

**Authors:** Sebastian Deorowicz

**Affiliations:** Faculty of Automatic Control, Electronics and Computer Science, Silesian University of Technology, Akademicka 16, 44-100 Gliwice, Poland

**Keywords:** Data processing, Software

## Abstract

The amount of data produced by modern sequencing instruments that needs to be stored is huge. Therefore it is not surprising that a lot of work has been done in the field of specialized data compression of FASTQ files. The existing algorithms are, however, still imperfect and the best tools produce quite large archives. We present FQSqueezer, a novel compression algorithm for sequencing data able to process single- and paired-end reads of variable lengths. It is based on the ideas from the famous prediction by partial matching and dynamic Markov coder algorithms known from the general-purpose-compressors world. The compression ratios are often tens of percent better than offered by the state-of-the-art tools. The drawbacks of the proposed method are large memory and time requirements.

## Introduction

In the recent years, genome sequencing has became a mature technology with numerous applications in the medicine. The instruments by Illumina (encountered to the 2nd generation of sequencers) produce majority of available data for little money (e.g., about one thousand of U.S. dollars for whole human genome sequencing). The sequenced reads are relatively short (up to a few hundreds of bases) but are of high quality. The 3rd generation instruments by PacBio or Oxford Nanopore can deliver much longer reads but unfortunately of much worse quality and at much lower throughput.

The estimations of the amount of genomic data and the related costs can be found in the studies like^1,2^. The presented huge numbers directly lead to the conclusion that in the near future just a storage and transfer of sequenced (and mapped) reads will consume a lot of money and could be a dominant factor in total costs related to sequencing. Therefore, it is not surprising that a lot of research was made to overcome this problem. The obvious first step was application of gzip, a general-purpose compressor. The about 3-fold reduction of files was remarkable, but the data deluge asked for more. The main problem of gzip is that it was designed mainly for textual data, or more precisely, for data with textual-like redundancy types. Unfortunately things like repetitions of parts of data at short distances are uncommon in read collections.

The next step was an invention of specialized algorithms taking into account types of redundancies specific for FASTQ files^3^. Some of the most important early results were presented in^4–7^. The details of the proposed algorithms were different, but in general the authors tried (with some exceptions) to compress the reads locally, i.e., not looking for the large-scale relations between the reads. The reason was rather simple and practical: the amounts of memory necessary to construct a dictionary data structure allowing to find overlaps between reads could be a few times larger than the input file size, e.g., hundreds of GB for human genomes. The improvement over gzip was, however, limited. For example, the best algorithms were able to reduce the space necessary for DNA bases about 5 times. This value should be compared to 4-fold reduction by simple spending 2 bits to distinguish among 4 valid bases. Moreover, it appeared that the compression of quality values was even more problematic.

This situation motivated researchers to look for alternatives. At the beginning, they focused just on the compression of bases. The key idea was to reorder the data to gather reads originating from close regions of genomes. This could seem as a loss of information, but as the original ordering of reads in a FASTQ file is usually more or less random one can argue that it is hard to say which of two random orderings is better (and even how we can define what “better” means here). The first notable attempt into this direction was described in^8^. The authors introduced a variant of the Burrows–Wheeler transform to find overlaps between reads. For human reads with 40-fold coverage they were able to spend about 0.5 bits per base, which was a significant improvement.

In the following years, other researchers explored the concept of using minimizers^9^, i.e., short, lexicographically smallest, substrings of sequences, to find reads from close regions. The key observation was that if two reads originate from the close regions of a genome their minimizers are usually the same. Thus, the reads can be grouped by their minimizers. In the first work following this idea^10^, for the mentioned human dataset it was sufficient to use about 0.3 bits per base. A similar result was obtained later in^11^. The possible gains were, however, limited by the fact that the reads identified to originate from the same genome region could span no more than two read lengths.

In^12^, it was shown how to group reads from a bit larger genome regions. Significantly better results were, however, obtained in three recent articles presenting HARC^13^, Spring^14^, and Minicom^15^. The attempts differ in details, but are based on similar ideas. The overlaps are found for much larger genome regions (in theory up to chromosome size) thanks to dictionary structures storing minimizers of parts of reads. What is also worth to mention, FaStore^12^ and Spring^14^ do not focus just on DNA bases and they can compress also the complete FASTQ files.

Together with improving the compression ratio for DNA symbols, the quality scores became responsible for a dominant part of the compressed archives. Therefore a number of works focused on this problem. One of the simplest strategies was to reduce the resolution of quality scores. Illumina in their HiSeq sequencers restricted the quality scores to eight values, and then in the NovaSeq instruments to just four values. The rational for these decisions was that the quality of sequencing is currently very good and the prices of sequencing are low. Therefore, if necessary, it is easier (and cheaper) to perform sequencing with a bit larger coverage than store high-resolution quality scores. The recent experiments suggest that reduction of quality score resolution has very little (if any) impact on the quality of variant calling. For example, in^12^ it was shown that even more aggressive reduction to just two quality values can be justified, at least in some situations. Moreover, there are several algorithms like QVZ^16,17^ and Crumble^18^ that perform advanced analysis of quality scores to preserve only the most important information.

In this article, we propose a novel compression algorithm for FASTQ files. The main novelty is in the compression of DNA bases, as for quality scores and read identifiers we follow similar strategies as in the state-of-the-art tools. Our algorithm, FQSqueezer, make use of the ideas from the prediction by partial matching (PPM)^19,20^ and dynamic Markov coder (DMC)^21^ general-purpose methods. A direct adaptation of the PPM-like strategy to sequencing reads would be, however, very hard and likely unsuccessful. There are at least four main reasons for that. First, in the ideal case, the PPM algorithm should construct a dictionary of all already seen strings of length up to some threshold, significantly larger than log_4_ (*genome*_*size*), which for human genomes seems to be unimplementable on workstations and even medium-sized servers. Second, the PPM algorithms often need many accesses to the main memory to compress a single symbol. For huge dictionaries this could result in a very slow processing (due to cache-misses). Third, sequenced data contain errors that should be corrected to refrain from expansion of the dictionary structures. Fourth, the PPM algorithms usually learn slowly, which is a good strategy for texts, but seems to be bad for genomic data.

To overcome these problems we designed a few fixed-*k* dictionaries for *k*-mers (*k*-symbol long substrings) found in the reads. Moreover, the dictionaries are organized in a way reducing the number of cache misses. We also estimate the probability of symbols occurrence much more aggressively, which results in significantly better compression (compared to classical PPM-like estimation). Finally, for the storage of *k*-mers in the dictionaries we perform some kind of error correction.

The proposed ideas has some similarity to earlier works. For example, some correction of bases was employed in AssembleTrie^22^. The authors used it, however, just to “synchronize” reads from both strands (forward and reverse). Formerly, in GeCo^23^ a similar correction was used to improve context determination in the field of genomic (complete genomes, chromosomes, contigs) data compression. The estimation of the probability of appearance of the current symbol based on *k*-mers of some size (much smaller than in our solution due to huge memory requirements of the picked dictionary implementation) was used in Fqzcomp^4^. Modeling the genomic data (genomes, chromosomes, contigs) as a Markov source was studied by Pinho *et al*.^24,25^. It is, however, worth to emphasis the differences between compression of sequencing reads and collections of longer genomic fragments, e.g., contigs, chromosomes, genomes. The longer (assembled) fragments are of much better quality than reads, so small differences between very similar fragments are usually due to variations between organisms and they are expected. In sequencing datasets, we usually work with reads originating from a single genome and the differences between very similar fragments are in majority due to sequencing errors. Some of them (minority) are also due to diploid structure of some genomes. Even in case of metagenomic studies, when the reads are from many genomes, a significant part of the differences are due to sequencing errors. Therefore, a compressor of sequencing data should be designed in a different way than a compressor of assembled genomes or its parts. They also should not be compared directly in practice as they were designed for solving different tasks.

In the present article, we significantly improved the mentioned ideas, as well as, proposed other techniques. The most important ones are: the organization of the huge dictionaries, design of the PPM-like estimation of probabilities, use of a custom DMC as the stage following the PPM, the technique for prediction and correction of sequencing errors, technique of ordering the reads and making use of shared prefixes. What is also important, we developed the complete FASTQ compressors.

The main asset of FQSqueezer is its compression ratio, usually much better than of the state-of-the-art competitors, i.e., FaStore^12^, Spring^14^, and Minicom^15^. Our tool has, however, also some drawbacks in terms of speed and memory usage. Namely, it is a few times slower than the mentioned competitors in compression and much slower in decompression.

## Results

### Tools and datasets

For the evaluation we used the state-of-the-art competitors, i.e., FaStore^12^, Spring^14^, and Minicom^15^. We resigned from testing some other good compressors like BEETL^8^, Orcom^10^, AssembleTrie^22^, and HARC^13^ as the previous works demonstrated that they perform worse than the picked tools. The older utilities are not competitive in terms of compression ratio, as was demonstrated in the recent papers^12,14^ (see also Table 1 for experiments with one of our datasets). Some of them are very fast, e.g., DSRC 2^7^. Nevertheless, in this article we focus mainly on the compression ratio.

**Table 1:** Comparison of compression ratios and running times of selected general-purpose compressors and FASTQ-specialized compressors.

Compressor	Comp. size [MB]	Comp. time [s]	RAM in comp. [MB]	Decomp. time [s]	RAM in decomp. [MB]
pigz	3,392	128	**10**	54	**2**
7z	2,710	2,438	5,592	220	71
zstd	3,335	828	48	35	4
brotli	3,186	6,214	100	78	4
DSRC 2	2,273	**55**	3,997	56	2,739
FQZcomp	1,990	287	283	385	581
NAF^27^	2,173	12,885	4,620	60	3,799
Spring	1,650	159	1,735	**24**	975
FQSqueezer	**1,511**	1,409	19,489	1,501	19,467

The dataset ERR532393_1 (complete FASTQ file) of size 9.64GB was used. The original ordering of reads were preserved.

The datasets for experiments were taken from the previous studies. They are characterized in Supplementary Table 1. In the main part of the article, we used 9 datasets but more results can be found in the Supplementary Worksheet. Unfortunately, some compressors do not support all the examined modes, which is a reason of lack of their results in some tables.

All experiments were run at workstation equipped with two Intel Xeon E5-2670 v3 CPUs (2 × 12 double-threaded 2.3 GHz cores), 256 GB of RAM, and six 1 TB HDDs in RAID-5. If not stated explicitly the programs were run with 12 threads.

### Compression of the bases

The most important part of the present work is the compression of bases. Therefore, in the first experiment we evaluated the tools in this scenario. The results for the single-end (SE) reads are given in Table 2. The ratios are in output bits per input base. As it is easy to observe, in the majority of cases FQSqueezer outperforms the competitors. For some datasets the gain is large. Nevertheless, for two datasets FQSqueezer loses to Minicom in the original-order-preserving (OO) mode. We investigated this situation a bit closer by checking whether the compression ratio will depend on the initial ordering of the reads. When we shuffled the reads prior to compression, the compression ratios for SRR1265495_1 and SRR1265496_1 changed significantly for all the examined compressors, i.e., 0.632 → 0.531 and 0.646 → 0.527 (Spring), 0.448 → 0.539 and 0.484 → 0.568 (Minicom), 0.506 → 0.472 and 0.517 → 0.487 (FQSqueezer). This shows that the initial ordering of the reads in these datasets is far from random and Minicom can benefit from this. In the mode allowing reordering of the reads (REO), FQSqueezer always wins. Moreover, the compression ratios are roughly twice better than in the OO mode for all the examined methods.

**Table 2:** Compression ratios for single-end reads.

Dataset	Size [Gbp]	Original ordering				Reordered				
		Spring	Minicom	FQSqueezer	Gain	FaStore	Spring	Minicom	FQSqueezer	Gain
ERR174310_1	20.97	0.696	0.857	**0.649**	7.4	0.593	0.408	0.589	**0.396**	3.1
ERR532393_1	3.58	0.667	0.647	**0.528**	22.5	0.482	0.433	0.410	**0.294**	39.3
SRR327342_1	0.95	0.524	0.538	**0.488**	7.4	0.267	0.158	0.166	**0.142**	11.3
SRR554369_1	0.17	0.441	0.509	**0.414**	6.6	0.494	0.240	0.307	**0.227**	5.6
SRR635193_1	1.47	0.697	0.702	**0.633**	10.0	0.333	0.267	0.289	**0.216**	23.9
SRR689233_1	1.48	0.449	0.442	**0.400**	10.6	0.248	0.193	0.187	**0.149**	25.5
SRR870667_1	7.48	1.460	1.364	**0.721**	89.3	0.722	1.292	1.212	**0.506**	42.6
SRR1265495_1	1.70	0.632	**0.448**	0.506	−11.4	0.368	0.500	0.319	**0.246**	29.7
SRR1265496_1	1.48	0.646	**0.484**	0.517	−6.4	0.391	0.507	0.352	**0.264**	33.4

Compression ratios are in output bits per base [bpb]. Best results are in bold. ‘Gain’ (expressed in %) is defined as: best competitor ratio divided by FQSqueezer ratio subtracted by 1. FaStore does not offer original ordering preserving.

The results for the paired-end (PE) reads can be found in Table 3. In the OO mode, FQSqueezer usually outperforms Spring significantly. Nevertheless, for the largest dataset it loses slightly. It is hard to say what is the reason. The situation for the REO mode is similar, but the gains are usually even larger than in the OO mode.

**Table 3:** Compression ratios for paired-end reads.

Dataset	Size [Gbp]	Orginal ordering			Reordered				
		Spring	FQSqueezer	Gain	FaStore	Spring	Minicom	FQSqueezer	Gain
ERR174310	41.93	**0.463**	0.474	−2.3	0.844	**0.317**	0.481	0.346	−8.3
ERR532393	7.15	0.623	**0.462**	34.7	0.694	0.505	0.471	**0.347**	35.8
SRR327342	2.08	0.490	**0.355**	38.0	—	0.307	—	**0.197**	55.7
SRR554369	0.33	0.322	**0.299**	7.7	0.652	0.222	0.293	**0.205**	8.4
SRR635193	2.94	0.562	**0.501**	12.2	0.500	0.339	0.419	**0.292**	16.0
SRR689233	2.96	0.367	**0.309**	18.8	0.350	0.239	0.257	**0.184**	30.2
SRR870667	12.60	1.070	**0.553**	93.3	—	0.928	—	**0.424**	119.0
SRR1265495	3.40	0.484	**0.373**	29.7	0.437	0.436	0.316	**0.228**	38.2
SRR1265496	2.96	0.500	**0.389**	28.4	0.479	0.449	0.344	**0.247**	39.0

Compression ratios are in output bits per base [bpb]. Best results are in bold. ‘Gain’ (expressed in %) is defined as: best competitor ratio divided by FQSqueezer ratio subtracted by 1. FaStore and Minicom do not support original ordering preserving. For two datasets (REO mode) we were unable to run FaStore and Minicom.

The most important drawbacks of FQSqueezer are, however, its time and space requirements (Table 4 and Supplementary Worksheet). In the compression, it is a few times slower than the competitors, but in the decompression the difference is larger. The reason is simple. FaStore, Spring, and Minicom need time to find overlapping reads that likely origin ate from close genome positions. Nevertheless, decoding of the matches between the overlapping reads is very fast. FQSqueezer can be classified as a PPM algorithm. In the decompression, the algorithms from this family essentially mimic the same work made in the compression, so the differences in compression and decompression times are negligible.

**Table 4:** Time and memory requirements for compression of SE reads in the reordering mode.

Dataset	Size [Gbp]	FaStore				Spring				Minicom				FQSqueezer			
		c-t	d-t	c-m	d-m	c-t	d-t	c-m	d-m	c-t	d-t	c-m	d-m	c-t	d-t	c-m	d-m
ERR174310_1	20.97	4,595	109	**6.4**	4.2	**1,815**	**103**	11.0	4.7	19,417	105	67.7	**4.0**	12,728	13,100	91.6	90.6
ERR532393_1	3.58	379	18	5.9	0.9	**150**	18	**3.0**	1.9	609	**17**	10.1	**0.7**	1,344	1,452	16.4	16.4
SRR327342_1	0.95	192	5	4.4	**0.3**	**35**	5	**1.4**	0.6	67	**3**	4.6	0.7	144	145	6.7	6.6
SRR554369_1	0.17	93	**1**	0.9	0.1	**7**	**1**	**0.8**	0.3	30	**1**	1.7	**0.0**	68	70	6.2	6.2
SRR635193_1	1.47	291	12	5.9	0.9	**77**	**10**	11.2	0.8	347	11	**5.4**	**0.3**	456	462	12.1	12.1
SRR689233_1	1.48	196	7	5.5	0.7	**64**	9	**1.6**	0.8	127	**5**	4.5	**0.3**	406	413	11.7	11.6
SRR870667_1	7.48	2,185	**64**	**5.8**	**3.0**	**757**	81	7.3	**3.0**	3,030	70	27.1	3.9	4,127	4,432	36.4	36.1
SRR1265495_1	1.70	483	**7**	5.7	0.6	**82**	12	**1.9**	1.6	203	**7**	5.1	**0.2**	658	685	13.2	13.1
SRR1265496_1	1.48	170	**6**	5.1	0.6	**81**	11	**1.8**	1.6	194	7	4.6	**0.2**	609	652	13.0	13.0

Column abbreviations: ‘c-t’ — compression time (in seconds), ‘c-m’ — RAM usage in compression (in GB), ‘d-t’ —decompression time (in seconds), ‘d-m’— RAM usage in decompression (in GB). Best results are in bold.

The case of memory usage is similar. The same dictionaries must be maintained by FQSqueezer in the decompression that are necessary in the compression. Moreover, to predict the successive symbols a lot of statistics must be collected. Nevertheless, without the applied correction mechanisms the occupied memory would likely be doubled.

The dictionaries are updated only at the synchronization points, i.e., after processing each FASTQ block of size 16 MB, so the number of threads has some impact on the compression ratio. The results in Table 5 show that reducing the number of threads from 12 to 1 we can gain 1–2% in ratio, but the processing would be significantly slower.

**Table 5:** Multithreding scalability of FQSqueezer for SRR327342_1 dataset.

No. threads	Original ordering		Reordered	
	Compression time [s]	Ratio [bpb]	Compression time [s]	Ratio [bpb]
1	1034.76	0.484	547.72	0.139
2	619.97	0.485	378.87	0.140
3	453.10	0.485	315.44	0.140
4	356.98	0.486	263.28	0.141
6	265.40	0.487	202.04	0.141
8	221.37	0.487	168.96	0.142
12	169.56	0.488	140.31	0.142
16	143.78	0.489	131.10	0.143
24	116.33	0.490	122.69	0.144

One of the parameters of FQSqueezer is the assumed genome size, which should be comparable to the true genome size in the sequencing experiment. The genome size is used to set the lengths of the *k*-mers stored in the dictionaries. In the last experiment in this part, we checked the impact of this parameter. The results presented in Table 6 show that declaring improper genome size deteriorates the compression ratio, but the differences are not large (In Supplementary Section 1.1 one can find some suggestion how to set this parameter).

**Table 6:** Impact of FQSqueezer declared genome size for SRR870667_1 dataset.

Declared genome size	Original ordering			Reordered		
	Time [s]	RAM [GB]	Ratio [bpb]	Time [s]	RAM [GB]	Ratio [bpb]
2	3,130	23.4	0.761	2,815	23.4	0,548
10	3,124	23.8	0.744	2,847	23.8	0.533
50	3,203	25.1	0.730	3,172	25.0	0.515
200	3,717	30.0	0.725	3,647	29.9	0.507
500	4,569	36.6	0.721	4,090	36.4	0.502
2000	4,867	68.0	0.722	4,958	67.8	0.500

Reference genome for this organism is of size 430 Mbp.

### Full FASTQ compressors

FQSqueezer is a FASTQ compressor, so we ran it for a few datasets to verify its performance in such scenario. There were only two competitors: FaStore and Spring as Minicom was designed just for bases. The results in Table 7 are for three modes. In the *lossless* mode, all data were preserved. In the *reduced* mode, the IDs were truncated to just the instrument name and the quality values were down-sampled to 8 levels (i.e., Illumina 8-level binning). In the *bases only* mode, only the bases are stored. The table presents only the sizes of the compressed archives, but the timings and memory occupation can be found in Supplementary Worksheet.

**Table 7:** Compression of complete FASTQ files.

Dataset	Input	Original ordering		Reordered		
		Spring	FQSqueezer	FaStore	Spring	FQSqueezer
**Lossless**						
ERR532393_1	9,642	1,649.6	**1,510.8**	1,840.4	1,738.9	**1,634.9**
SRR327342_1	2,813	435.4	**428.2**	504.1	471.5	**468.0**
SRR870667_1	18,555	3,913.6	**3,091.2**	3,683.6	4,067.2	**3,342.5**
ERR532393	19,284	3,200.6	**2,903.6**	3,602.1	3,299.3	**3,032.0**
SRR327342	5,986	954.6	**904.1**	—	987.2	**944.3**
SRR870667	32,402	6,060.6	**5,093.6**	—	6,201.6	**5,345.0**
**Reduced**						
ERR532393_1	9,642	917.5	**827.8**	978.4	814.4	**723.5**
SRR327342_1	2,813	178.7	**172.1**	208.2	135.5	**131.2**
SRR870667_1	18,555	2,583.7	**1,824.0**	2,151.7	2,444.0	**1,620.3**
ERR532393	19,284	1,814.9	**1,616.5**	2,058.3	1,729.8	**1,515.9**
SRR327342	5,986	413.4	**368.9**	—	366.6	**328.4**
SRR870667	32,402	3,914.7	**2,994.2**	—	3,698.5	**2,790.2**
**Bases only**						
ERR532393_1	9,642	298.0	**236.7**	215.5	193.3	**132.1**
SRR327342_1	2,813	62.1	**59.2**	31.6	18.8	**17.0**
SRR870667_1	18,555	1,364.2	**673.6**	674.8	1,207.5	**473.2**
ERR532393	19,284	556.6	**416.5**	620.6	451.5	**313.5**
SRR327342	5,986	127.1	**97.8**	—	79.7	**52.7**
SRR870667	32,402	1,684.5	**871.6**	—	1,461.0	**667.2**

The dataset names suffixed “_1” denote SE data. The remaining are PE files. All numbers are sizes in MBs. The best results are in bold.

The experiment confirmed the advantage of FQSqueezer in terms of compression ratio. Nevertheless, our compressor is slower and needs more memory than the competitors. It is interesting to note that in the lossless modes both Spring and FQSqueezer give smaller archives when they do not permute the reads. This is caused by the large cost of compression of IDs that are not so similar for subsequent reordered reads. In the remaining modes, the cost of ID storage is negligible, so the reordering modes win.

## Discussion

In the article, we presented a novel compression algorithm for FASTQ files. Its architecture was motivated by the PPM and DMC general-purpose compressors. Nevertheless, significant amount of work was necessary to make it possible to adapt these two approaches for genomic data. First, it was crucial to prepare specialized data structures for statistics gathering, with care of fast memory accesses (i.e., reduction of cache misses). Second, some dedicated correction of bases was implemented for better prediction and reduction of memory usage. Third, a special approach was necessary to efficiently store paired reads. Fourth, the DMC-like mechanism for aggressive estimation of symbol occurrence probabilities was proposed.

The experiments show advantage of FQSqueezer in terms of compression ratio for the majority of datasets. The differences between our tool and the state-of-the-art competitors were sometimes quite large. Nevertheless, for the largest paired-end dataset we perform slightly worse than Spring. This phenomenon deserves further investigation.

The most important drawbacks of FQSqueezer are slow processing and large memory consumption. These features are typical for PPM-based algorithms. Nevertheless, some work to reduce these drawbacks is probably possible. For example, the two most important components responsible for slow processing are looking for approximate matches and queries for incomplete *k*-mers. In the future work, it should be possible to attack at least these two problems, e.g., try to minimize the number of queries to the dictionary data structures without deterioration of the compression ratios.

For those, who would find the memory and time requirements prohibitive for application of the proposed tool in real pipelines, FQSqueezer could be seen as an attempt into better estimation of the entropy present in sequencing data. It should also be possible to implement some of the concepts present in FQSqueezer in the more practical FASTQ compressors to improve their compression ratios.

## Methods

### Basic definitions

For clarity of description of the algorithm let us assume that DNA sequences $x={x}_{1}{x}_{2}\ldots {x}_{r}$ contain symbols from alphabet {A,C,G,T,N}. The *length* (*size*) of a sequence is the number of elements it is composed of. A *substring* can be obtained from a sequence by removing (possibly 0) symbols from the beginning and the end. The notation *x*_*i,j*_ means a substring ${x}_{i}{x}_{i+1}\ldots {x}_{j}$. A *k-mer* is a sequence of length *k*. A *canonical k*-mer is lexicographically smaller of a *k*-mer and its reverse complement.

### Basic description of the algorithm

FQSqueezer is a multi-threaded algorithm, but for simplicity of presentation we will start from a single-threaded variant. Our tool accepts both single-end (SE) and paired-end (PE) reads. The reads can be stored in the original ordering (OO) or can be reordered (REO). In the reordering mode, the reads are initially sorted according to the DNA sequence (first read of a pair in the PE mode).

The input FASTQ files (or sorted files in the REO mode) are loaded in blocks of size 16 MB. The reads from a single block (pair of blocks in the PE mode) are compressed one by one (or pair by pair). The read ID and quality values are compressed using rather standard means (similarly like in the top existing FASTQ compressors). The details are described at the end of this section. Below, we will focus just on the DNA symbols.

### Compression of bases

In the compression of a SE read (or first read of a pair), we process the bases from the beginning of a read position by position. For each base we determine the statistics of occurrences of *k*-mers ending at this base in the already processed part of the input data. To this end, we maintain a few dictionaries: *D*_e_, *D*_p_, *D*_s_, and *D*_b_ that store numbers of occurrences of: *e*-mers, *p*-mers, *s*-mers, and *b*-mers, respectively, where $e < p < s < b$. The details of the organization of the dictionaries are given in Supplementary Section 1.1.

At the beginning we will discuss the OO-SE mode. In general, the longest possible, but no longer than *b*–1 symbols, context (substring preceding the current symbol in a read) is taken to predict the current symbol. Then, the symbol is encoded using these predictions (as well as some other properties of a read and the current position) with a use of a range coder.

**Figure 1:**
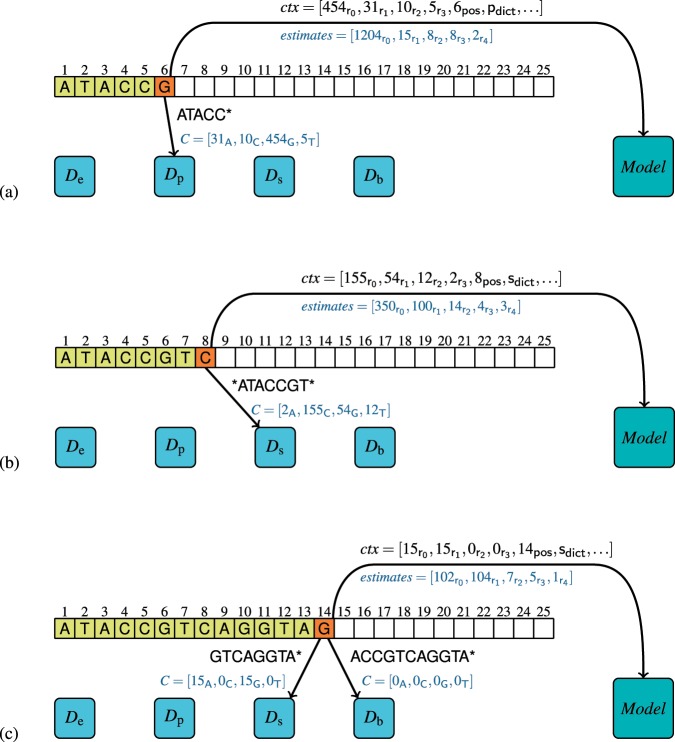
Illustration of compression of a single read in a single-end, original ordering mode. The subfigures (**a**–**c**) show how the estimation of probabilities for entropy coding is performed for some symbols.

Let us follow the example given in Fig. 1 assuming the sizes of *k*-mers: *e* = 4, *p* = 6, *s* = 9, *b* = 12. The subfigures (a)–(c) show how the probabilities of appearance of each symbol from the alphabet {A,C,G,T,N} are estimated for some symbols of a read. In Fig. 1a, the 6th symbol (orange cell in the figure) is encoded. We consult the *D*_p_ dictionary (as there are too few already-processed-read symbols to use *D*_s_ or *D*_b_) for statistics of appearance of all *p*-mers ATACC*, where “*” represents any symbol. The answer (blue font in the figure) is that: ATACC**A** appeared 31 times, ATACC**C**—10 times, ATACC**G**—454 times, ATACC**T**—5 times. Then, we reorder the alphabet symbols according to this statistics (for simplicity let us assume that for equal counters the symbols are ordered lexicographically): G (rank 0), A (rank 1), C (rank 2), T (rank 3), N (rank 4). In the next step, we check in the *Model* dictionary how many times for such ordered counters, at the 6th position, when the statistics are from the *D*_p_ dictionary the current symbol was the one with rank 0, 1,…. (In fact we use more items that define the query to the *Model* dictionary, but for simplicity of presentation we omit them here. More details are given in Supplementary Section 1.2.) The answer is that 1204 times the symbol of rank 0 was encoded, 15 times the symbol of rank 1, and so on. The frequencies 1204, 15, 8, 8, 2 are then used by the range coder to encode the current symbol.

The situation presented in Fig. 1b is a bit different. Here we are at the 8th position in a read so we are able to look for statistics in *D*_s_ dictionary. Nevertheless, this dictionary stores statistics for 9-mers and we can construct only 8-mers from the already processed symbols. Therefore, the dictionary is asked for *ATACCGT* 9-mers, where “*” represents any symbol. The answer is that: *ATACCGT**A** appeared 2 times, *ATACCGT**C**—155 times, *ATACCGT**G**—54 times, *ATACCGT**T**—12 times. Then, we ask the *Model* dictionary and obtain estimates: 350 for rank-0 symbol (C in this situation), 100 for rank-1 symbol (G), 14 for rank-2 symbol (T), 4 for rank-3 symbol (A), 3 for rank-4 symbol (N). These estimates are used for encoding the current symbol using the range coder.

In Fig. 1c, we show the processing of the 14th symbol. Now, the number of processed symbols is sufficient to use *D*_b_ dictionary for statistics of occurrence of 12-mers ACCGTCAGGTA*. Unfortunately, the answer is that no such 12-mer has been seen so far. Therefore, we use the *D*_s_ dictionary for GTCAGGTA* and obtain statistics: 15 for A, 0 for C, 15 for G, 0 for T. Then we use the *Model* dictionary and see that the estimates for the current symbol are: 102 for rank-0 symbol (A), 104 for rank-1 symbol (G), 7 for rank-2 symbol (C), 5 for rank-3 symbol (T), 1 for rank-4 symbol (N). As previously, these values can be used to encode the current symbol by the range coder.

When a symbol is encoded, it is checked whether it looks like a sequencing error. Let us assume that the statistics from the *D*_b_ dictionary are $C=[{0}_{{\rm{A}}},{5}_{{\rm{C}}},{0}_{{\rm{G}}},{0}_{{\rm{T}}}]$ and the current symbol is A. The dominance of C symbols suggests that A is a sequencing error. Therefore, we encode A at this position but replace A by C in the read, which has impact on the processing of the next symbols in the read. To assume that we have a sequencing error the most frequent symbol should have counter at least 3 and the current symbol should have counter 0.

In the REO mode, the prefix of size *p* of a SE read (or first read of a pair in the PE mode) is encoded in a different way. Roughly speaking, we treat it as a 2*p*-bit unsigned integer and encode the difference between it and the integer representing the prefix of the previous read. Using the same read as in the above example, we pick the 6-mer ATACCG and convert it to 12-bit integer using mapping: 00_2_ for A, 01_2_ for C, 10_2_ for G, 11_2_ for T. Therefore the read prefix is represented as ${001100010110}_{2}={790}_{10}$. Let us also assume the previous read prefix was ATACAT, which translates to ${001100010011}_{2}={787}_{10}$. Therefore the current read prefix is encoded as a difference $790-787=3$. The differences are usually small numbers which can be encoded efficiently using a range coder. The suffix is compressed in the same way as in the OO mode.

In the PE modes, the first read of a pair is compressed exactly in the same way as in the SE mode. The processing of the second read is a bit more complex, but it is the same in the OO and REO modes. Initially we try to predict some *b*-mer of the second read. To this end, we use a dictionary *M*_b_ that stores pairs of minimizers of read pairs seen so far. Quite often this allows to encode the *b*-mer of the second read in an efficient way. Then, we store the substrings of the read following and preceding this *b*-mer. If we were unable to predict the minimizer of the second read, we store the read in the same way as a SE read in the OO mode.

**Figure 2:**
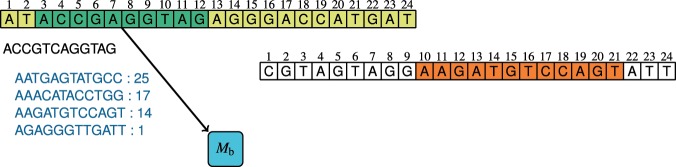
Example of prediction of some *b*-mer of the paired read from the statistics of occurrences of pairs of minimizers in PE reads.

Figure 2 shows an example how the pair of minimizers are used to predict some of the second read *b*-mer. At the beginning a minimizer, ${m}_{1}={\rm{ACCGAGGTAG}}$, in the first read is found (green cells). Then, we look in the *M*_b_ dictionary which minimizers in the second read appeared together with *m*_1_ in the past. We obtain four candidates ordered by the number of appearances. Then for each of the candidates we check whether it appears in the second read. In our example, we found AAGATGTCCAGT (orange cells). Thus, we encode just the rank of the candidate in the ordered list of candidates, i.e., 2 (we count from 0) and the position of the candidate in the second read, i.e., 10.

After processing a read the dictionaries *D*_e_, *D*_p_, *D*_s_, *D*_b_, and *M*_b_ for a case of PE reads, are updated.

For clarity of presentation, the above description of bases compression is a bit simplified. For example, in practice we work on canonical *k*-mers, a second correction mechanism (rarely used) is also employed, more than a single pair of minimizers is stored in *M*_b_ dictionary, the *Model* dictionary is indexed with a use of some function of the mentioned (and other) properties of the current position in a read, a dynamic Markov coder-like mechanism is used in the *Model* dictionary to provide estimated probabilities. A discussion of all of this can be found in Supplementary Section 1.2.

### Compression of IDs

In the lossless mode, IDs are compressed similarly as in the state-of-the-art compressors, like Spring or FaStore. The ID of each read is tokenized (the separators are non-alphanumerical characters). Then the tokens are compared with the tokens of the previous read. If the tokens at corresponding positions contain numerical values, the difference between the integers is calculated and stored. Otherwise the corresponding tokens are compared as strings. If they are equal we just store a flag. In the opposite case, we store a mismatch flag and compare the tokens character by character storing the result of comparison and (if necessary) the letter from the current read. It can also happen that the list of tokens differ significantly, i.e., they are of different length or the corresponding tokens are of different type. In this situation, the ID is stored character by character.

FQSqueezer offers also two lossy modes. In the first one, it preserves just the instrument name (first part of the ID). These names are organized in a move-to-front list^26^ and the position of the current ID at the list is encoded. If the current instrument name is absent form the list, it is encoded explicitly. In the last possible mode, IDs are discarded.

### Compression of quality scores

The quality scores can be compressed in five modes allowing different number of values: 96, 8, 4, 2, none. If the input FASTQ file has already reduced resolution of quality scores (e.g., 4 for Illumina NovaSeq sequencers), no conversion is necessary. Otherwise, in the lossy mode the necessary resolution reduction is made by the compressor. The encoding is made using contexts containing the position in a read and 2 (96-value alphabet), 6 (8-value alphabet), 9 (4-value alphabet), or 10 (binary alphabet) previous scores.

### Implementation details

The implementation is in the C++14 programming language. Most of the dictionaries are implemented as hash tables with linear probing for collision handling. To reduce delays caused by cache misses we make use of software prefetching. The multithreading is implemented using the native C++ threads. The FASTQ blocks of size 16 MB are split into as many parts as the number of threads. Each thread processes its part independently and the global dictionaries are available only for querying. At the synchronization points the threads update the global dictionaries. Nevertheless, the threads update different parts of the dictionaries so they can operate in parallel.

## Supplementary information

Supplementary info.

Supplementary info2.

## Data Availability

The source code of the application is available at https://github.com/refresh-bio/fqsqueezer.
