# Author Correction: FQSqueezer: *k*-mer-based compression of sequencing data

**DOI:** 10.1038/s41598-020-60472-x

**Published:** 2020-02-21

**Authors:** Sebastian Deorowicz

**Affiliations:** Faculty of Automatic Control, Electronics and Computer Science, Silesian University of Technology, Akademicka 16, 44-100 Gliwice, Poland

Correction to: *Scientific Reports* 10.1038/s41598-020-57452-6, published online 17 January 2020

The Supplementary info2 file originally published with this Article was incorrectly provided as a LaTeX class file. The correct Supplementary info2 file now accompanies the Article.

