# Supplementary material for: FQSqueezer: k-mer-based compression of sequencing data
Source: Sci Rep. 2020 Jan 17;10:578. doi: 10.1038/s41598-020-57452-6 (PMC6969201; doi:10.1038/s41598-020-57452-6)
Supplement: Supplementary file 1 — Supplementary info. [file 41598_2020_57452_MOESM1_ESM.pdf]

# Supplementary material for article: FQSqueezer: $k$-mer-based compression of sequencing data

Sebastian Deorowicz

November 25, 2019

## Contents

**1 Algorithmic details** **2**
1.1 Data structures organization . . . 2
1.2 Algorithmic details . . . 3
1.2.1 SE reads — original ordering . . . 3
1.2.2 SE reads — reordered . . . 5
1.2.3 PE reads — original ordering . . . 5
1.2.4 PE reads — reordered . . . 5

**2 Examined programs** **6**

**3 Datasets** **10**

**4 Environment** **11**

# 1 Algorithmic details

## 1.1 Data structures organization

The dictionaries are organized in various ways. The $D_{\mathrm{p}}$ dictionary is a vector of 2-bit integers of size $4^p$. It is indexed by an integer representation of a $p$-mer. Each 2-bit field stores a number of occurrences of a related $p$-mer, saturated at 3. When a $p$-mer is added to the dictionary the counters are incremented for both $p$-mer and its reversed complement. The concept of treating the $k$-mers and its reverse complement similarly is present in may previous works, e.g. [3, 2].

The $D_{\mathrm{s}}$ dictionary is a collection of $4^{s-10}$ hash tables representing canonical $s$-mers and the counters (no. of occurrences of each $s$-mer). When looking for (or inserting to) $s$-mer $x$ in the dictionary, a hash table from the collection is identified by the substring $x_{3,s-8}$. Each 32-bit entry of a hash table contains: 4 bits for $x_{1,2}$, 16 bits for $x_{s-7,s}$, and 12 bits for the counter. A hash function calculates its value just using $x_{3,s-2}$, i.e., the kernel of an $s$-mer. Such an organization allows to make use of software prefetching mechanism to reduce delays related to cache misses. Moreover, the $s$-mers sharing their $(s-1)$ starting symbols are at close positions, which also reduces the number of cache misses. The maximal value of a counter is $2^{12}-1$, which can be saturated for repetitive genomes. Therefore, the counter incrementation is a bit tricky. Counter values $v$ not exceeding $2^{11}$ are always incremented. The larger values are incremented with probability $1/(v-2^{11})$. Thus, the large counts are approximate, but the maximal representable value is much larger.

The $D_{\mathrm{b}}$ dictionary is organized in a similar way as $D_{\mathrm{s}}$, but here we have a collection of $4^{b-13}$ hash tables. The hash table is identified by an integer representation of $x_{3,b-11}$. The 32-bit entries are composed of: 4 bits for $x_{1,2}$, 22 bits for $x_{b-10,b}$, and 6 bits for the counter. The incrementation of counter value $v$ larger than 8 is with probability $1/2(v-8)$.

The compressor maintains global dictionaries $D_{\mathrm{p}}$, $D_{\mathrm{s}}$, and $D_{\mathrm{b}}$ that can be queried in parallel by separate threads. The update of these dictionaries is after processing the complete 16 MB blocks (in fact for the 1st, 2nd, ..., 99th block it is more frequent, i.e., $100 - \mathit{block\_number}$ times for a block). It is made by all threads as each thread updates different hash tables from a collection (or different part of vector $D_{\mathrm{p}}$). The delayed update means, however, that the recently processed $k$-mers are absent from the global dictionaries. To overcome this problem, each thread stores also small local dictionaries $D_{\mathrm{s}}^{\mathrm{local}}$ and $D_{\mathrm{b}}^{\mathrm{local}}$ for representation of $s$-mers and $b$-mers seen in this thread from the last update of the global dictionaries. This local dictionaries are hash tables with 64-bit entries.

The $D_{\mathrm{e}}$ dictionary is a hash table storing statistics of occurrences of all substrings of length from 1 to $e$.

In the PE mode, we need also $M_{\mathrm{b}}$ dictionary. It is a hash table containing 128-bit entries storing two $b$-mers that are signatures (variant of a minimizer, defined as in [2]) of some parts of reads and the counter stored at $64-2b$ bits. More precisely, for each pair of reads $x$ and $y$ we determine several signatures, i.e.,

- $\underline{x^1}$, $\underline{x^2}$, $\underline{x^3}$ — the signatures in the first, second, and third part of $x$,
- $\underline{y^1}$, $\underline{y^2}$, $\underline{y^3}$ — as above for $y$,
- $\underline{x^{23\mathrm{rc}}}$ and $\underline{y^{23\mathrm{rc}}}$ — the signatures in the reverse-complemented second and third part of $x$, and $y$.

Then, we add the following pairs to $M_{\mathrm{b}}$: $\langle\underline{x^1},\underline{y^1}\rangle$, $\langle\underline{x^1},\underline{y^3}\rangle$, $\langle\underline{x^1},\underline{x^{23\mathrm{rc}}}\rangle$, $\langle\underline{x^2},\underline{y^1}\rangle$, $\langle\underline{x^2},\underline{y^3}\rangle$, $\langle\underline{x^3},\underline{y^1}\rangle$, $\langle\underline{x^3},\underline{y^3}\rangle$, $\langle\underline{y^1},\underline{x^1}\rangle$, $\langle\underline{y^1},\underline{x^3}\rangle$, $\langle\underline{y^1},\underline{y^{23\mathrm{rc}}}\rangle$, $\langle\underline{y^2},\underline{x^1}\rangle$, $\langle\underline{y^2},\underline{x^3}\rangle$, $\langle\underline{y^3},\underline{x^1}\rangle$, $\langle\underline{y^3},\underline{x^3}\rangle$. The local dictionary $M_{\mathrm{b}}^{\mathrm{local}}$ is maintained in the similar way as local variants of other dictionaries.

The choice of parameters $f$, $p$, $s$, $b$ has some impact on the compression ratios. They should be adjusted to the genome size of the sequenced organism (or a sum of genome sizes in

Table 1: Determination of dictionary parameters

| Genome size [Mbp] | $e$ | $f$ | $p$ | $s$ | $b$ |
|---|---|---|---|---|---|
| $(0, 1]$ | 1–9 | 9 | 14 | 17 | 19 |
| $(1, 4]$ | 1–9 | 9 | 15 | 18 | 20 |
| $(4, 16]$ | 1–9 | 10 | 15 | 18 | 21 |
| $(16, 64]$ | 1–9 | 11 | 16 | 18 | 23 |
| $(64, 256]$ | 1–9 | 12 | 17 | 20 | 24 |
| $(256, 1024]$ | 1–9 | 12 | 17 | 21 | 26 |
| $(1024, 4096]$ | 1–9 | 13 | 18 | 21 | 27 |
| $(4096, 16384]$ | 1–9 | 14 | 18 | 22 | 27 |
| $(16384, 65536]$ | 1–9 | 15 | 18 | 22 | 27 |

metagenomic experiments). Therefore, the user should specify the genome size, which is used to set the parameters as shown in Table 1. If the genome size is unknown, it roughly can be guessed from the FASTQ file size and the sequenced coverage.

## 1.2 Algorithmic details

There are 4 different modes in which FQSqueezer can be run: (*i*) for single-end (SE) reads with preserving the original ordering of the reads, (*ii*) for SE reads without preserving the original ordering of the reads, (*iii*) for paired-end (PE) reads with preserving the original ordering of the reads, (*iv*) for PE reads without preserving the original ordering of the reads. Most of the algorithmic details are the same in the modes, so we will start the description from the simplest SE order-preserving mode.

### 1.2.1 SE reads — original ordering

The first $f$ symbols of a read are compressed using the statistics of occurrence of up to $e$ previous symbols. This value is 1 at the beginning of compression and quite fast grows to 9 (when there are sufficient amount of statistics). The processing of successive symbols is as follows. We maintain 3 pairs of $k$-mers:

- $(p-1)$-mers: $z_{\mathrm{p}}^{\mathrm{unc}}$ and $z_{\mathrm{p}}$, where the first contains recently seen $p-1$ symbols and the second is a copy of $z_{\mathrm{p}}^{\mathrm{unc}}$ in which some symbols could be changed by our correction mechanism,
- $(s-1)$-mers: $z_{\mathrm{s}}^{\mathrm{unc}}$ and $z_{\mathrm{s}}$ defined as above for $s-1$ recent symbols,
- $(b-1)$-mers: $z_{\mathrm{b}}^{\mathrm{unc}}$ and $z_{\mathrm{b}}$ defined as above for $b-1$ recent symbols.

At the beginning of the processing of a read the $k$-mers are incomplete.

Let us assume that we are at the $i$th position in a read $x$. First, we try to obtain as accurate predictions of the current symbol as possible. To this end, if $s < i$ we ask $D_{\mathrm{b}}$ and $D_{\mathrm{b}}^{\mathrm{local}}$ for statistics of occurrences of $z_{\mathrm{b}}$ followed by A, C, G, or T. If $z_{\mathrm{b}}$ is complete, the queries are straightforward. Otherwise, to collect the statistics we fill $z_{\mathrm{b}}$ (at the beginning) by all possible combinations of bases, which can be costly (in terms of time). In case of no success, we try also the same for $z_{\mathrm{b}}^{\mathrm{unc}}$.

If we were unable to collect any statistics and $p < i$, we try the similar procedure for $z_{\mathrm{s}}$ and $D_{\mathrm{s}}$, $D_{\mathrm{s}}^{\mathrm{local}}$. Moreover, we use this step also in situation in which for more than one base the counters obtained from querying $D_{\mathrm{b}}$ achieve 63, the maximal counter value.

If necessary (no success), the search is performed for $z_{\mathrm{p}}$ and $D_{\mathrm{p}}$.

If we were not able to find any statistics in the previous stages we perform more aggressive search. If $b \leq i$, we try to collect the statistics for $z_{\mathrm{b}}$ and $D_{\mathrm{b}}$ for approximate matches (with 1 mismatch). Unfortunately, this means dozens of queries. In case of no success and $s \leq i$, we try the same for $z_{\mathrm{s}}$ and $D_{\mathrm{s}}$. Finally, if necessary, we use also $z_{\mathrm{p}}$ and $D_{\mathrm{p}}$.

If after this search procedure we do not have any statistics, we encode the current symbol taking into account from 1 to 9 previous symbols (exactly like for the read prefix). The situation is more interesting if we were able to collect some statistics. We form a context for encoding of the current symbol. The context components are:

1. *pos* — current position in a read,
2. *pattern* — sorted counters of occurrences of symbols,
3. *cnt_lev* — origin of statistics, one of:
   - $z_{\mathrm{b}}$ or $z_{\mathrm{b}}^{\mathrm{unc}}$ and $D_{\mathrm{b}}$ or $D_{\mathrm{b}}^{\mathrm{local}}$,
   - $z_{\mathrm{s}}$ or $z_{\mathrm{s}}^{\mathrm{unc}}$ and $D_{\mathrm{s}}$ or $D_{\mathrm{s}}^{\mathrm{local}}$,
   - $z_{\mathrm{p}}$ or $z_{\mathrm{p}}^{\mathrm{unc}}$ and $D_{\mathrm{p}}$,
   - $z_{\mathrm{b}}$ or $z_{\mathrm{b}}^{\mathrm{unc}}$ and $D_{\mathrm{b}}$ or $D_{\mathrm{b}}^{\mathrm{local}}$ with additional information from $z_{\mathrm{s}}$ and $D_{\mathrm{s}}$,
4. *accuracy* — number of accurate predictions of the actual at previous 8 positions,
5. *top_symbol* — the symbol with the highest counter value,
6. *correction* — one of:
   - there was a correction in the most recent half of $z_{\mathrm{b}}$,
   - there was a correction in the former half of $z_{\mathrm{b}}$,
   - the statistics are from the approximate queries,
   - none of the above.

The actual context is constructed from the context components in the way motivated by the dynamic Markov coder [1]. We allow 7 levels of contexts. In the parentheses we give the number of times the context can be used before it is split into more detailed ones (i.e., before we go to the next level):

1. context is empty (1),
2. context contains (32):
   - *cnt_lev*,
   - *pos* rounded to 16 if we are not at the beginning of a read (values smaller than $b$ are not rounded) and not close to the end of a read (last 5 positions in a read are stored explicitly),
   - two largest counters down-sampled to: 30 values (if the counters are from the $D_{\mathrm{p}}$ queries) or 10 values (otherwise),
   - the remaining counters down-sampled to: 10 values (if the counters are from the $D_{\mathrm{p}}$ queries) or 5 values (otherwise),
3. context contains additionally: *accuracy* and *correction* (64),
4. if the counters are not obtained from the $D_{\mathrm{p}}$, the two largest counters are down-sampled to 15 values (64),

5. if the counters are not obtained from the $D_\mathrm{p}$, the two largest counters are down-sampled to 30 values and the remaining counters to 10 values, otherwise the two smallest counters are down-sampled to 30 values (128),

6. context contains additionally: *top_symbol* (512),

7. the positions rounded to 16 are now rounded to 8.

At the end of processing of a current position, we try to correct (just to make better representation in the dictionaries) the current symbol. If $i < b$ we do nothing with the exception for a case in which the current symbol is N, as we update it to A. For $i \geq b$ we alternatively run two correction procedures. First is applied if the counters are from inspecting the $D_\mathrm{b}$ or $D_\mathrm{b}^\mathrm{local}$. If the counter value for the current symbol is 0 and the most frequent symbol has counter 3 or more we make a substitution. Second is applied in case of no statistics or if the statistics are from the $D_\mathrm{p}$. Moreover, to apply this correction the current coverage (estimated from the number of updates to $D_\mathrm{p}$) must be at least 7. We try to change one of the previous 6 symbols to find a match in the $D_\mathrm{b}$.

As a simple time optimization at the beginning of processing of a read we check whether it is a copy of the previous read. We store this information and do not process copied reads position by position.

### 1.2.2 SE reads — reordered

In the REO mode, the reads are initially split into 256 bins according to the first 4 symbols (Ns are treated as Ts). Then the reads are read from bins and sorted in the main memory before further processing.

The only difference in the compression from the OO mode is handling of the read prefix. We treat the first $p$ symbols as an $2p$-bit unsigned integer $t$. Let $t^\mathrm{prev}$ be such value for the previous read. We check in $D_\mathrm{p}$ the counter $c$ for $t$. We encode $c$ and then determine (in $D_\mathrm{p}$) the number of $p$-mers between $t^\mathrm{prev}$ and $t$ with counter equal $c$ and store this number.

A special care is necessary for Ns in the read prefix. They are replaced by Ts for the conversion to the integers, but a special flag is stored to tell whether such substitutions took place in the present read. If necessary the substitutions are stored to allow perfect reconstruction of the prefix.

### 1.2.3 PE reads — original ordering

The compression of PE reads in the OO mode is similar to the compression of SE reads. The first read of a pair is compressed exactly in the same way. The second read also can be compressed in the same way, but fortunately, quite often we can do much better. To this end, we determine 4 signatures of the first read, i.e., form the 1st, 2nd, 3rd, 4th part of the read. For each signature we ask $M_\mathrm{b}$ for the paired candidate signatures. We merge, the lists and pick the top 16 candidates. Then in the second read of a pair we look for the candidates. In case of success, we just store the position of the found signature at the candidate list and the position of the signature in the second read. Then, we store the parts after and before the signature in the second read using the same way as in the SE mode.

### 1.2.4 PE reads — reordered

The compression of PE reads in the REO mode is a mix of the compression of SE-REO and PE-OO modes. The first read of a pair is stored as in the SE-REO mode. The second one is processed as in the PE-OO mode.

## 2 Examined programs

The following programs were used in the experimental part. The running parameters are also given.

- FaStore v. 0.8:
  FaStore was downloaded from `https://github.com/refresh-bio/FaStore`.
  - SE compression of bases:
    `./fastore_compress.sh --max --in in.fastq --threads 12 --out out.fastore`
  - SE decompression of bases:
    `./fastore_decompress.sh --in out.fastore --threads 12 --out decomp.fastq`
  - PE compression of bases:
    `./fastore_compress.sh --max --in in1.fastq --pair in2.fastq --threads 12 --out out.fastore`
  - PE decompression of bases:
    `./fastore_decompress.sh --in comp.fastore --threads 12 --out decomp.fastq`
  - SE compression in reduced mode:
    `./fastore_compress.sh --reduced --threads 12 --in in.fastq --out out.fastore`
  - SE compression in lossless mode:
    `./fastore_compress.sh --lossless --threads 12 --in in.fastq --out out.fastore`
  - PE compression in reduced mode:
    `./fastore_compress.sh --reduced --threads 12 --in in1.fastq --pair in2.fastq --out out.fastore`
  - PE compression in lossless mode:
    `./fastore_compress.sh --lossless --threads 12 --in in1.fastq --pair in2.fastq --out out.fastore`

- Spring (version from date 2018-Jul-11):
  Spring was downloaded from `https://github.com/shubhamchandak94/Spring`.
  - SE compression of bases, original ordering
    `.spring -c --no-ids --no-quality -i in.fastq -o comp.spring -t 12`
  - PE compression of bases, original ordering
    `./spring -c --no-ids --no-quality -i in1.fastq in2.fastq -o comp.spring -t 12`
  - SE compression of bases, reordered
    `./spring -c --no-ids --no-quality -i in.fastq -o comp.spring -t 12 -r`
  - PE compression of bases, reordered
    `./spring -c --no-ids --no-quality -i in1.fastq in2.fastq -o comp.spring -t 12 -r`
  - SE compression, lossless, original ordering
    `./spring -c -q lossless -t 12 -o output.spring -i in.fastq`

- SE compression, lossless, reordered
  ```
  ./spring -c -q lossless -t 12 -o output.spring -i in.fastq -r
  ```
- PE compression, lossless, original ordering
  ```
  ./spring -c -q lossless -t 12 -o output.spring -i in1.fastq in2.fastq
  ```
- PE compression, lossless, reordered
  ```
  ./spring -c -q lossless -t 12 -o output.spring -i in1.fastq in2.fastq -r
  ```
- SE compression, reduced, original ordering
  ```
  ./spring -c -q ill_bin -t 12 -o output.spring -i in.fastq --no-ids
  ```
- SE compression, reduced, reordered
  ```
  ./spring -c -q ill_bin -t 12 -o output.spring -i in.fastq -r --no-ids
  ```
- PE compression, reduced, original ordering
  ```
  ./spring -c -q ill_bin -t 12 -o output.spring -i in1.fastq in2.fastq --no-ids
  ```
- PE compression, reduced, reordered
  ```
  ./spring -c -q ill_bin -t 12 -o output.spring -i in1.fastq in2.fastq -r --no-ids
  ```

• Minicom (version from date 2018-Dec-27):
Minicom was downloaded from https://github.com/yuansliu/minicom

- SE compression of bases, reordered
  ```
  ./minicom -r in.fastq -t 12
  ```
- SE compression of bases, original ordering
  ```
  ./minicom -r in.fastq -t 12 -p
  ```
- PE compression of bases, reordered
  ```
  ./minicom -1 in1.fastq -2 in2.fastq -t 12
  ```

• FQSqueezer v. 0.1:
FQSqueezer was downloaded from https://github.com/refresh-bio/fqsqueezer

- SE compression of bases, original ordering
  ```
  ./fqs-0.1 e -im n -qm n -om o -gs <genome_size> -s -t 12 -out comp.fqs in.fastq
  ```
- SE compression of bases, reordered
  ```
  ./fqs-0.1 e -im n -qm n -om s -gs <genome_size> -s -t 12 -out comp.fqs in.fastq
  ```
- PE compression of bases, original ordering
  ```
  ./fqs-0.1 e -im n -qm n -om o -gs <genome_size> -p -t 12 -out comp.fqs in1.fastq in2.fastq
  ```
- PE compression of bases, reordered
  ```
  ./fqs-0.1 e -im n -qm n -om s -gs <genome_size> -p -t 12 -out comp.fqs in1.fastq in2.fastq
  ```

- SE compression, lossless, original ordering
  ```
  ./fqs-0.1 e -gs <genome_size> -im o -qm o -om o -s -t 12 -out comp.fqs in.fastq
  ```
- SE compression, lossless, reordered
  ```
  ./fqs-0.1 e -gs <genome_size> -im o -qm o -om s -s -t 12 -out comp.fqs in.fastq
  ```
- PE compression, lossless, original ordering
  ```
  ./fqs-0.1 e -gs <genome_size> -im o -qm o -om o -p -t 12 -out comp.fqs in1.fastq in2.fastq
  ```
- PE compression, lossless, reordered
  ```
  ./fqs-0.1 e -gs <genome_size> -im o -qm o -om s -p -t 12 -out comp.fqs in1.fastq in2.fastq
  ```
- SE compression, reduced, original ordering
  ```
  ./fqs-0.1 e -gs <genome_size> -im i -qm 8 -om o -s -t 12 -out comp.fqs in.fastq
  ```
- SE compression, reduced, reordered
  ```
  ./fqs-0.1 e -gs <genome_size> -im i -qm 8 -om s -s -t 12 -out comp.fqs in.fastq
  ```
- PE compression, reduced, original ordering
  ```
  ./fqs-0.1 e -gs <genome_size> -im i -qm 8 -om o -p -t 12 -out comp.fqs in1.fastq in2.fastq
  ```
- PE compression, reduced, reordered
  ```
  ./fqs-0.1 e -gs <genome_size> -im i -qm 8 -om s -p -t 12 -out comp.fqs in1.fastq in2.fastq
  ```

- 7z v. 16.02
  - Comparison of general-purpose and FASTQ-specialized compresors
    ```
    ./7z a -mx9 -mmt12 out.7z in.fastq
    ```
- brotli v. 1.0.7
  - Comparison of general-purpose and FASTQ-specialized compresors
    ```
    ./brotli -9 -k -o out.brotli in.fastq
    ```
- DSRC v. 2.02
  - Comparison of general-purpose and FASTQ-specialized compresors
    ```
    ./dsrc c -m1 -t12 -k in.fastq out.dsrc
    ```
- FQZcomp v. 4.6
  - Comparison of general-purpose and FASTQ-specialized compresors
    ```
    ./fqzcomp -s6+ -e in.fastq out.fqz
    ```
- NAF v. 1.1.0
  - Comparison of general-purpose and FASTQ-specialized compresors
    ```
    ./ennaf --temp-dir ./tmp -22 --fastq -o out.naf in.fastq
    ```

- pigz v. 2.3.4
  - Comparison of general-purpose and FASTQ-specialized compresors
    `./pigz -p 12 -9 in.fastq`

- zstd v. 1.4.4
  - Comparison of general-purpose and FASTQ-specialized compresors
    `./zstd -9 -k in.fastq`

# 3 Datasets

Table 2: Datasets used in the experiments. The paired-end datasets were obtained from two files, i.e., '_1' and '_2'. Descriptions are from https://www.ebi.ac.uk

| Dataset | Read len. | No. reads | Description |
|---|---|---|---|
| ERR174310_1<br>ERR174310_2 | 101<br>101 | 207,579,467<br>207,579,467 | Illumina HiSeq 2000 paired end sequencing; whole genome sequence data for the CEPH 1463 family |
| MH0001_081026_clean.1<br>MH0001_081026_clean.2 | 44<br>44 | 11,640,674<br>11,640,358 | Human gut |
| SRR1265495_1<br>SRR1265495_2 | 101<br>101 | 16,828,175<br>16,828,175 | Illumina HiSeq 2000 sequencing; GSM1375986: Normal lung tissue 1017-NOR; Homo sapiens; RNA-Seq |
| SRR1265496_1<br>SRR1265496_2 | 101<br>101 | 14,634,195<br>14,634,195 | Illumina HiSeq 2000 sequencing; GSM1375987: Normal lung tissue 1079-NOR; Homo sapiens; RNA-Seq |
| SRR1294116 | 101 | 45,974,333 | Illumina HiSeq 2000 sequencing; GSM1395310: human ES cell line H9 (ucla_h9); Homo sapiens; RNA-Seq |
| SRR1294122 | 101 | 39,666,314 | Illumina HiSeq 2000 sequencing; GSM1395316: human ES cell line UCLA6 (ucla6); Homo sapiens; RNA-Seq |
| SRR327342_1<br>SRR327342_2 | 63<br>75 | 15,036,699<br>15,036,699 | Illumina Genome Analyzer II sequencing |
| SRR445718 | 100 | 32,943,665 | Illumina HiSeq 2000 sequencing; GSM896803: Oocyte #1-C1; Homo sapiens; RNA-Seq |
| SRR445719 | 100 | 30,690,825 | Illumina HiSeq 2000 sequencing; GSM896804: Oocyte #1-C2; Homo sapiens; RNA-Seq |
| SRR445724 | 100 | 50,896,528 | Illumina HiSeq 2000 sequencing; GSM896809: 2-cell #1-C1; Homo sapiens; RNA-Seq |
| SRR445726 | 100 | 49,180,773 | Illumina HiSeq 2000 sequencing; GSM896811: 2-cell #2-C1; Homo sapiens; RNA-Seq |
| SRR490961 | 100 | 49,127,668 | Illumina HiSeq 2000 sequencing; GSM922146: 4-cell embryo#1 -Cell#1; Homo sapiens; RNA-Seq |
| SRR490962 | 100 | 45,583,212 | Illumina HiSeq 2000 sequencing; GSM922147: 4-cell embryo#1 -Cell#2; Homo sapiens; RNA-Seq |
| SRR490976 | 100 | 33,261,944 | Illumina HiSeq 2000 sequencing; GSM922161: 8-cell embryo#1 -Cell#4; Homo sapiens; RNA-Seq |
| SRR554369_1<br>SRR554369_2 | 100<br>100 | 1,657,871<br>1,657,871 | Illumina Genome Analyzer IIx paired end sequencing; Whole genome sequence of salinity tolerant biocontrol strain of Pseudomonas P17 |
| SRR635193_1<br>SRR635193_2 | 54<br>54 | 27,265,881<br>27,265,881 | Illumina Genome Analyzer IIx paired end sequencing; pooled amnion from 5 term birth placentas |
| SRR689233_1<br>SRR689233_2 | 90<br>90 | 16,407,945<br>16,407,945 | Illumina HiSeq 2000 paired end sequencing; GSM1080195: mouse oocyte 1; Mus musculus; RNA-Seq |
| SRR870667_1<br>SRR870667_2 | 108<br>74 | 69,230,235<br>69,230,235 | Illumina Genome Analyzer IIx paired end sequencing; Theobroma cacao Matina 1-6 genomic Illumina reads v3 |
| ERR532393_1<br>ERR532393_2 | 100<br>100 | 35,752,873<br>35,752,873 | Illumina HiSeq 2000 paired end sequencing; assembly for metagenome |

# 4 Environment

The computer used in the tests was of the following configuration:

- 2 Intel Xeon E5-2670 v3 CPUs, 12 double-threaded cores per CPU, each clocked at 2.3 GHz,
- 256 GiB RAM,
- 6 HDDs of size 1 TiB each in RAID-5 configuration, `hdparm -t` reported buffered read speed 528.45 MB/s.

For compilation we used G++ v. 7.2.0. The machine was running Debian 9.3.1 x86-64 OS.

## References

[1] Cormack, G. V. & Horspool, R. N. S. Data compression using dynamic Markov modelling. *The Computer Journal* **30**, 541–550 (1987).

[2] Deorowicz, S., Kokot, M., Grabowski, S. & Debudaj-Grabysz, A. KMC 2: Fast and resource-frugal k-mer counting. *Bioinformatics* **31**, 1569–1576 (2015).

[3] Pinho, A. J., Neves, A. J. R., Ferreira, P. J. S. G. Inverted-repeats-aware finite-context models for DNA coding. In: Proc. of the 16th European Signal Processing Conference. IEEE Computer Society, Lausanne, Switzerland (2008).
